# Human Gallbladder Cancer Organoids

**DOI:** 10.1111/cpr.70211

**Published:** 2026-08-27

**Authors:** Dongxi Xiang, Ting Gong, Yinjie Zhu, Bin Jia, Chen Zhang, Ning Tang, Weiping Wang, Yalong Wang, Laiping Zhong, Yanmei Zou, Jian Zhang, Zunqiang Zhou, Ligang Xing, Changchun Zhou, Dongyuan Zhu, Zengjun Liu, Gang Chen, Xiaoguang Wang, Zhan Wang, Aina He, Yonggang Wang, Zebing Liu, Jiabei Wang, Feng Xu, Ganglong Gao, Xiaonan Kang, Shuai Gong, Xianming Kong, Chunyan Dong, Aijin Ma, Gengming Niu, Guiying Wei, Hongling Zhao, Xiongxiong Lu, Jie Cao, Tongbiao Zhao, Zizhen Zhang, Yingbin Liu

**Affiliations:** ^1^ State Key Laboratory of Systems Medicine for Cancer, Shanghai Cancer Institute Shanghai Jiao Tong University School of Medicine Shanghai China; ^2^ Department of Biliary‐Pancreatic Surgery Renji Hospital Affiliated to Shanghai Jiao Tong University School of Medicine Shanghai China; ^3^ Department of Gastrointestinal Surgery Renji Hospital Affiliated to Shanghai Jiao Tong University School of Medicine Shanghai China; ^4^ Department of Oncology Tianjin Medical University General Hospital Tianjin China; ^5^ Department of Urology Renji Hospital Affiliated to Shanghai Jiao Tong University School of Medicine Shanghai China; ^6^ Lung Cancer Department Tianjin Cancer Hospital Tianjin China; ^7^ Department of Hepatobiliary Surgery, Liver Transplant Center, Union Hospital, Tongji Medical College Huazhong University of Science and Technology Wuhan China; ^8^ School of Health Science and Engineering University of Shanghai for Science and Technology Shanghai China; ^9^ Department of Pharmacology and Pharmacy, Li Ka Shing Faculty of Medicine The University of Hong Kong Hong Kong China; ^10^ Guangzhou National Laboratory Guangzhou China; ^11^ Department of Stomatology, Oralmaxillofacial Head and Neck Surgery, Huashan Hospital Fudan University Shanghai China; ^12^ Department of Oncology, Tongji Hospital, Tongji Medical College Huazhong University of Science and Technology Wuhan China; ^13^ Department of Medical Oncology Fudan University Shanghai Cancer Center Shanghai China; ^14^ Department of Surgery Shanghai Jiao Tong University Affiliated Sixth People's Hospital Shanghai China; ^15^ Department of Radiation Oncology, Shandong Cancer Hospital and Institute Shandong First Medical University, Shandong Academy of Medical Science Jinan China; ^16^ Biobank, Cancer Research Center, Shandong Cancer Hospital and Institute Shandong First Medical University, Shandong Academy of Medical Sciences Jinan China; ^17^ Rare Tumors Department, Shandong Cancer Hospital and Institute Shandong First Medical University and Shandong Academy of Medical Sciences Jinan China; ^18^ Jiaxing Key Laboratory of Basic Research and Clinical Translation on Orthopedic Biomaterials, Department of Orthopaedics The Second Affiliated Hospital of Jiaxing University Jiaxing China; ^19^ Department of Hepatobiliary Surgery Affiliated Hospital of Jiaxing University Jiaxing China; ^20^ Department of Medical Oncology, Changzheng Hospital Second Military Medical University Shanghai China; ^21^ Department of Oncology Shanghai Jiao Tong University Affiliated Sixth People's Hospital Shanghai China; ^22^ Department of Pathology Renji Hospital Affiliated to Shanghai Jiao Tong University School of Medicine Shanghai China; ^23^ Department of Hepatobiliary Surgery, the First Affiliated Hospital of USTC, Division of Life Sciences and Medicine University of Science and Technology of China Anhui China; ^24^ Department of Medical Oncology, Shanghai Gongli Hospital Second Military Medical University Shanghai China; ^25^ Department of Biobank Renji Hospital Affiliated to Shanghai Jiao Tong University School of Medicine Shanghai China; ^26^ Division of Gastroenterology and Hepatology Renji Hospital Affiliated to Shanghai Jiao Tong University School of Medicine Shanghai China; ^27^ Collaborative Research Center for Biomedicines Shanghai University of Medicine and Health Sciences Shanghai China; ^28^ Department of Oncology, East Hospital Affiliated to Tongji University Tongji University School of Medicine Shanghai China; ^29^ Beijing Technology and Business University Beijing China; ^30^ Shanghai OneTar Biomedicine Shanghai China; ^31^ Beijing Institute for Stem Cell and Regeneration Medicine Beijing China; ^32^ Standards Committee, Chinese Society for Cell Biology Shanghai China; ^33^ Department of General Surgery, Pancreatic Disease Center, Ruijin Hospital Shanghai Jiao Tong University School of Medicine Shanghai China; ^34^ Department of Hepatology Surgery Renji Hospital Affiliated to Shanghai Jiao Tong University School of Medicine Shanghai China; ^35^ Key Laboratory of Organ Regeneration and Reconstruction, State Key Laboratory of Stem Cell and Reproductive Biology, Institute of Zoology Chinese Academy of Sciences Beijing China

## Abstract

This guideline provides standardized requirements and quality control measures for human gallbladder cancer organoids, supporting their reliable application in research and precision medicine. It aims to promote methodological consistency, facilitate international collaboration, and accelerate translational research and clinical implementation.

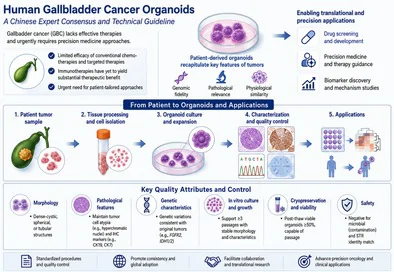

The clinical management of patients with gallbladder cancer remains challenging [1]. Conventional chemotherapeutic regimens and targeted therapies have demonstrated limited efficacy, whereas immunotherapies have yet to yield substantial therapeutic benefit in this maliganancy [1]. Consequently, their is an urgent, unmet clinical need for precision‐medicine approaches tailored to individual gallbladder cancer patients. Over the past decade, significant advances have been achieved in the developed of patient‐derived organoids as in vitro models that recapitulate key features of human tumors [2]. Patient‐derived organoids preserve the physiological, pathological and genomic characteristics of their parental tumour tissues, providing unprecedented opportunities for translational research, therapeutic discovery and precision oncology [2]. The ‘Human Gallbladder Cancer Organoids’ guideline represents an integral component of Human Cancer Organoids Guidelines series in China, which was jointly developed by experts from the Chinese Society for Cell Biology and its affiliated branches [3]. The Chinese version of this guideline was officially released on 28 October 2024. This standard document establishes comprehensive frameworks covering the terminologies, technical requirements, evaluation criteria and quality control procedures for the genaration and application of human gallbladder cancer organoids. The publication of the English version of this standard aims to facilitate global dissemination and adoption of standardized practices for human gallbladder cancer organoids. By promoting methodological consistency and quality assurance, this guideline is expected to support international collaboration, accelerate translational research, and advance the integration of GBCUnidentified organoid into biomedical research, drug development, and precision clinical applications.

## 1 Scope

This document specifies the general requirements, technical requirements, testing methods, inspection rules, instructions for use, labelling, transportation and storage requirements for human gallbladder cancer organoids.

## 2 Normative References

The provisions of the following documents constitute essential requirements of this document through normative references. For dated references, only the edition cited edition applies. For undated references, the latest edition of the referenced document (including all amendments) applies.

*Pharmacopoeia of the People's Republic of China* (2025 Edition, Part III).

## 3 Terms and Definitions

The following terms and definitions apply to this document.

### 3.1 Organoids

Three‐dimensional (3D) in vitro cultures derived from stem cells or progenitor cells that process self‐organisation and self‐renewal capabilities, comprise organ‐specific cell types and recapitulate the structural features and specific functions of the original tissue [4].

### 3.2 Human Gallbladder Cancer Organoids

Organoids established from gallbladder tumour cells from patients with pathologically confirmed gallbladder cancer, which recapitulate the biological characteristics of original gallbladder cancer tissues.

### 3.3 Organoid Passage

The process of dissociating established organoids into smaller fragments or single cells via physical, chemical or biological methods, followed by inoculation and in vitro expansion under the same culture conditions as the original culture.

### 3.4 Organoid Cryopreservation

The process of preserving organoids under low temperature in an inactive state to maintain their cellular compositions, gene expression profiles, and functional properties.

### 3.5 Organoid Thawing

The process by which cryopreserved organoids recover growth capability and metabolic activity from an inactively state.

## 4 Abbreviations

The following abbreviations apply to this document.

3D: Three dimension

DAB: Diaminobenzidine

DMSO: Dimethyl sulfoxide

DNA: Deoxyribonucleic acid

PBS: Phosphate‐buffered saline

PCR: Polymerase chain reaction

STR: Short tandem repeat

H&E: Haematoxylin and eosin

## 5 General Requirements

### 5.1 Raw Materials

5.1.1 The acquisition of raw materials shall comply with nationally recognized ethical standards and applicable local laws and regulations [5, 6].
5.1.2 According to the intended application, donor evaluation criteria shall be established for the research and production of human gallbladder cancer organoids.

### 5.2 Process and Information Management

5.2.1 Critical factors that may affect product quality during raw material acquisition, preparation, testing, transportation and storage shall be documented. Unidentified. A unique identification system shall be established to ensure full‐process traceability.
5.2.2 The minimum retention period for relevant records shall be clearly defined to ensure the integrity, confidentiality, and security of documentation.

## 6 Technical Requirements

### 6.1 Morphology

Human gallbladder cancer organoids exhibit diverse morphological features including dense‐cystic, spherical or tubular shapes under optical microscopy.

### 6.2 Pathological Features

6.2.1 The organoid cells shall retain the atypical characteristics of tumour cells from the original tumour tissue, including hyperchromatic nuclei, abnormal mitotic figures and altered nuclear‐cytoplasmic ratio.
6.2.2 Immunohistochemical testing for relevant biomarkers shall be performed on organoids, and the results shall be generally consistent with those of the original tumour tissues. The tested markers include, but are not limited to, CK19 and CK7.

### 6.3 Genetic Characteristics

Genetic variation analysis shall be performed on organoids, and the results shall be highly consistent with those of the original tumour tissue. Genes tested include, but are not limited to, *FGFR2* and *IDH1/2*.

### 6.4 In Vitro Culture and Growth

6.4.1 Human gallbladder cancer organoids derived from gallbladder cancer patient tissues or cells shall be capable of being passaged for at least three generations after the initial culture in vitro.
6.4.2 Organoids after passage shall be capable of reconstructing new organoid structures in vitro, with morphology, pathological characteristics, and genetic characteristics consistent with those of the parental organoids.

### 6.5 Survival Rate of Organoids

The viable number of thawed human gallbladder cancer organoids after cryopreservation shall not be less than 50% of the number before freezing. The surviving organoids shall retain the ability for in vitro passaged.

### 6.6 Microorganisms

Organoids shall be negative for fungi, bacteria, mycoplasma and viral contamination testing.

### 6.7 STR

The identity of organoids shall be consistent with that of the donor tissue based on STR analysis.

## 7 Testing Methods

### 7.1 Morphology

Organoids shall be cultured under three‐dimensional in vitro conditions and observed using optical microscopy.

### 7.2 Pathological Features

Representative testing methods are provided in Supplementary Annex A.

### 7.3 Genetic Characteristics

Representative testing methods are provided in Supplementary Annex B.

### 7.4 In Vitro Culture and Growth

Three‐dimensional cultured organoids shall be photographed using optical microscopy. Scale bars shall be included for diameter measurement and quantitative analysis.

### 7.5 Survival Rate of Organoids

Representative testing methods are provided in Supplementary Annex C.

### 7.6 Microorganisms

7.6.1 Fungi and Bacteria

The ‘1101 Sterility Inspection Method’ specified in *Pharmacopoeia of the People's Republic of China* (2020 Edition, Part III) shall be followed.

7.6.2 Mycoplasma

The ‘3301 Mycoplasma Inspection Method’ specified in *Pharmacopoeia of the People's Republic of China* (2020 Edition, Part III) shall be followed.

7.6.3 Exogenous Viral Factors

The ‘3302 Exogenous Viral Factors Inspection Method’ specified in *Pharmacopoeia of the People's Republic of China* (2020 Edition, Part III) shall be followed.

### 7.7 STR

Representative testing methods are provided in Supplementary Annex D.

## 8 Instructions for Use

The instructions shall include at least the following information:

**Table:** 

(a) Organoid code;	(h) Transportation conditions;
(b) Passage number;	(i) Contact information;
(c) Organoid quantity;	(j) Usage instructions;
(d) Production date;	(k) Execution standard number;
(e) Batch number;	(l) Production address;
(f) Manufacturing organization;	(m) Postal code;

*Note:* Endotoxin results should be provided upon user request.

## 9 Labelling

The labels shall include at least the following information:

**Table:** 

(a) Organoid code;	(d) Batch number;
(b) Passage number;	(e) Manufacturing organization;
(c) Organoid quantity;	(f) Production date.

## 10 Transportation and Storage

### 10.1 Transportation

10.1.1 The transportation mode and conditions shall be selected according to the intended requirements for human gallbladder cancer organoids to ensure the preservation of their biological characteristics, safety, stability and functional properties.
10.1.2 The transportation of human gallbladder cancer organoids shall consider, but not be limited to, factors including organoid characteristics, organoid containers, transportation routes, transportation conditions, transportation equipment, transportation modes, potential risks and necessary protective measures.
10.1.3 Control measures for transportation conditions shall include, but not be limited to, temperature range control, vibration management, contamination prevention, equipment performance assurance and appropriate packaging.
10.1.4 Relevant inspection reports and technical guidance documents shall be provided upon user request.
10.1.5 Package shall be inspected during transportation. When necessary, additional cryogenic materials (e.g., dry ice and liquid nitrogen) shall be supplemented to maintain the required transportation temperature.

### 10.2 Storage [7, 8]

10.2.1 Optimised cryopreservation protocols and procedures shall be applied to minimise damage to human gallbladder cancer organoids during cryopreservation and thawing, ensuring minimal impact on their normal biological functions.
10.2.2 Cryopreservation records for human gallbladder cancer organoids shall be documented, including but not limited to:

**Table:** 

(a) Organoid code;	(e) Freezing date;
(b) Batch number;	(f) Cryoprotectant composition;
(c) Organoid quantity;	(g) Name of the operator.
(d) Passage number;
10.2.3 10.2.3 The storage conditions for human gallbladder cancer organoids should be documented, including but not limited to:
  - Storage conditions;
  - Storage date;
  - Storage duration;
  - Name of the operator.

## Author Contributions

Dongxi Xiang, Ting Gong, Yinjie Zhu, Bin Jia, Chen Zhang, Ning Tang, Weiping Wang, Yalong Wang, Laiping Zhong, Yanmei Zou, Jian Zhang, Zunqiang Zhou, Ligang Xing, Changchun Zhou, Dongyuan Zhu, Zengjun Liu, Gang Chen, Xiaoguang Wang, Zhan Wang, Aina He, Yonggang Wang, Zebing Liu, Jiabei Wang, Feng Xu, Ganglong Gao, Xiaonan Kang, Shuai Gong, Xianming Kong, Chunyan Dong, Aijin Ma, Gengming Niu, Guiying Wei, Hongling Zhao, Xiongxiong Lu, Jie Cao, Tongbiao Zhao, Zizhen Zhang, Yingbin Liu critically read and revised the manuscript.

## Funding

This work was supported by the National Natural Science Foundation of China (32130036, 82573430, 82173358, 62202304, 82403651), the Shanghai Municipal Health Commission (2024ZZ2032, 2025ZZ1023), the Science and Technology Commission of Shanghai Municipality (23141901000), the School of Medicine, Shanghai Jiao Tong University (02.101005.001.29.38A) and the Laboratory of Early Prevention and Treatment for Regional High‐Frequency Tumor (GKE‐KF202307, GKE‐KF202404).

## Ethics Statement

The authors have nothing to report.

## Consent

The authors have nothing to report.

## Supporting information


**Annex A (Informative):** Organoid histopathology testing (paraffin embedding method).
**Annex B (Informative):** Organoid gene mutation testing.
**Annex C (Informative):** Quantification of organoid survival rates (Calcein‐AM staining method).
**Annex D (Informative):** Organoid authentication by STR profiling.

## Data Availability

The data that support the findings of this study are available on request from the corresponding author. The data are not publicly available due to privacy or ethical restrictions.
